# Conditional disease‐free survival rates and their associated determinants in patients with epithelial ovarian cancer: A 15‐year retrospective cohort study

**DOI:** 10.1002/cnr2.1416

**Published:** 2021-05-05

**Authors:** Vahid Ebrahimi, Abolfazl Khalafi‐Nezhad, Fatemeh Ahmadpour, Zahra Jowkar

**Affiliations:** ^1^ Department of Biostatistics, School of Medicine Shiraz University of Medical Sciences Shiraz Iran; ^2^ Department of Hematology, Medical Oncology and Stem Cell Transplantation School of Medicine, Shiraz University of Medical Sciences Shiraz Iran; ^3^ Department of Obstetrics and Gynecology, School of Medicine Shiraz University of Medical Sciences Shiraz Iran; ^4^ Oral and Dental Disease Research Center, Department of Operative Dentistry, School of Dentistry Shiraz University of Medical Sciences Shiraz Iran

**Keywords:** conditional disease‐free survival, histology, ovarian cancer

## Abstract

**Background:**

The most common type of ovarian cancer (OC) is epithelial ovarian cancer (EOC) which is the most lethal gynecologic malignancy in adult women.

**Aim:**

This study aimed to determine the conditional disease‐free survival (CDFS) rates and their associated determinants in patients with EOC.

**Methods and results:**

The clinical and demographic data of 335 patients with confirmed EOC at Motahari Clinic (Shiraz, Iran) were retrospectively reviewed and analyzed. Traditional DFS (TDFS) and CDFS were calculated using the Kaplan–Meier method and cumulative DFS estimates, respectively. To evaluate the effects of the prognostic determinants on the DFS of the patients, a multiple covariate Cox analysis using the landmarking method was applied.

The 1‐ and 3‐year TDFSs were 81.1% and 47.0%, respectively, and decreased over time. At baseline, a higher stage tumor and endometrioid histology were associated with a higher risk of recurrence when compared to stage I and other histological subtypes, respectively. The hazard of recurrence for older women (age ≥55 years) was approximately twice and three times more than that of women aged <45 years at 1‐ and 3‐year landmark time points, respectively.

**Conclusion:**

The age at diagnosis, defined by a cut‐off of 55 years, was a prognostic factor for the CDFS of EOC women. Moreover, patients with advanced‐stage EOC (ASEOC) (stages III and IV) and endometrioid histology had poorer CDFSs compared to those with early‐stage EOC (ESEOC) (stages I and II) and other histological types. In ESEOC patients with age at diagnosis of >55 years, CDFS gradually decreased in 3 years after remission which should be considered for follow‐up care decision‐making.

## 1 INTRODUCTION

Ovarian cancer (OC) is considered the seventh most common cancer among women worldwide. The most common type of OC is epithelial ovarian cancer (EOC) which is the most lethal gynecologic malignancy in adult women.^1^ The estimated overall 5‐year survival rate for early‐stage epithelial ovarian cancer patients (ESEOC: stages I and II) and patients with advanced‐stage epithelial ovarian cancer (ASEOC: stages III and IV) varies between 55% and 92% and 18% and 30%, respectively.^2^, ^3^

The statistical measures made at the time of diagnosis are traditionally used for evaluating the survival rate. In this regard, two types of traditionally used survival estimates in previous studies are overall survival (OS) and disease‐free survival (DFS) which are reported from the time of diagnosis and remission, respectively.^3^, ^4^, ^5^, ^6^ Despite providing important information for both clinicians and patients, such analyses appear to be of less value for patients who survive a period of time after their initial diagnosis and treatment.^7^ Although most patients with EOC attain remission, most of them eventually relapse. Follow‐up attentions including the close monitoring of CA‐125 levels, imaging tests, and physical exams are necessary and important in affecting disease outcomes.^8^, ^9^ A more accurate estimate for these patients is conditional DFS (CDFS) which considers changes in the patients' recurrence risks over time.^7^

Moreover, a recent clinical trial demonstrated that initiating chemotherapy for EOC patients with CA‐125 elevation and without symptoms did not offer any more benefits than delaying treatment until the emergence of clinical evidence of disease recurrence.^10^ Additionally, the early treatment of the relapsed EOC patients based on the rise of CA‐125 levels alone has resulted in the earlier deterioration of their quality of life.^10^ In these situations, using traditional DFS (TDFS) may not provide realistic and optimistic information about the recurrence risk for EOC patients, and CDFS estimates might offer more accurate information regarding the risk of recurrence so that the best‐informed decisions about the patients' follow‐up care can be made.^7^

Today, the risk of recurrence from the time of treatment or surgery can be computed using the available instruments for predicting the recurrence risk of EOC. CDFS estimates are based on the definition of conditional survival probability. These estimates incorporate the dynamic change in the survival risk over time. Therefore, they have been recognized as a more meaningful indicator of the survival probability of patients who have an initial survival period.^7^, ^11^

The median DFS was reported to be 2.54 years (range: 0.03‐9.96 years) among patients with OC in a previous study. Moreover, 3‐year DFS was 48.2%.^12^

The stage and histological type can affect the OS of patients with EOC. Moreover, age ≥ 60 years was correlated with poorer overall survival than age <60 years in a previous study.^13^ Another study showed that chemotherapeutic regimens/cycles and tumor grade and stage were independent prognostic factors for early‐stage EOC. Moreover, no significant differences were observed between 5‐year OS and histological types.^14^

Considering the insufficient data in the literature, this study aimed to determine the conditional disease‐free survival (CDFS) rates and their associated determinants in Iranian patients with EOC.

## 2 METHODS

### 2.1 Sampling population

This cohort study retrospectively reviewed the clinical and demographic data of women newly diagnosed with EOC at Motahari Clinic (a single tertiary referral hospital, Shiraz, Iran) from 2001 to 2016. The study protocol was approved by the Ethics Committee for Research of Shiraz University of Medical Sciences (Protocol# IR.SUMS.REC.1393.8910) and was conducted in compliance with the Declaration of Helsinki. Patients with nonepithelial ovarian cancer, tumors with an unknown or not applicable stage, borderline histology tumor, undefined histological types, without pathology confirmation, and lost to follow‐up were excluded. Besides, the patients who did not provide signed informed consent for the analysis of their medical records and those with nonanalyzable data (deficit in data) were also excluded.

The age at diagnosis, marriage status, child‐bearing (parity), tumor histology, and the FIGO (International Federation of Gynecology and Obstetrics) stage were considered for the analysis. The FIGO classification was used for disease staging and histological grading.^6^ Stages I and II were considered as ESEOC and stages III and IV were regarded as ASEOC. As the first‐line chemotherapy regimen, platinum‐paclitaxel was given to patients eligible for chemotherapy (except for patients with stage IA and grade I disease). According to the opinion of the physician and the tolerance of patients to the side effects of chemotherapy, three to six cycles of chemotherapy were applied.

### 2.2 Analysis of the survival time

The primary endpoint was DFS. The time interval between the initial remission date and the recurrence date or the last contact was considered as the DFS time. Patients without diagnosed recurrent EOC during the follow‐up period were censored at the end of the study. The date of diagnosis was defined as the date of the primary surgery in patients without cytology or the date of the first positive cytology. The date on which no evidence of disease was found by the oncologist was considered as the date of remission. If this information was missing, the date of the first negative surgical or imaging result or the date of the first factor indicating no evidence of disease such as normal levels of CA‐125 was used for defining the date of remission. When none of these criteria were available, 1 month after the date of chemotherapy completion or (if no chemotherapy was given) the date of the primary surgery was used. Recurrence was defined as a return of clinical symptoms on follow‐up after the patient has been in remission for a while. The date of recurrence was defined based on a process similar to the one applied for the date of remission. If the date on which an oncologist first diagnosed any signs of recurrence was available, it was considered as the date of recurrence. The date of the first positive surgical or imaging result, the initiation date of chemotherapy/radiation, or the date of the first evidence indicating disease recurrence such as elevated CA‐125 levels after being disease‐free for a while was used as the recurrence date. The TDFS rates were calculated by nonparametric Kaplan–Meier (NPKM) estimates and compared using the log‐rank test.^15^

The simultaneous impact of various patients' characteristics on the DFS was analyzed using the multiple covariate Cox‐adjusted proportional hazards (PH) model and employing the Breslow method for the ties. After assessing the PH assumption (using the goodness‐of‐fit testing approach based on Schoenfeld residuals), the findings were interpreted using hazard ratios (HRs). The confidence intervals (CIs) for the HRs were computed based on Wald's test of the Cox‐adjusted PH regression parameters. The landmark analysis method was applied with six landmark time points (at baseline and after 1, 2, 3, 4, and 5 years from the baseline).

### 2.3 Conditional disease‐free survival ( **CDFS** )

CDFS estimates were directly calculated from traditional NPKM disease‐free survival estimates. CDFS is defined as the probability of staying disease‐free for an additional number of years (t_2_) provided that a patient has already been in remission for t_1_ number of years. It is expressed as CDFS (t_2_|t_1_). The exponential version of Greenwood's formula (see Appendix A) was used to compute the CDFS estimates with a confidence interval (CI) of 95%.^16^ The changes in DFS over time were evaluated by comparing the estimations of 1‐ and 3‐year CDFSs (CDFS (1|x) and CDFS (3|x)) at 1, 2, 3, 4, and 5 years after attaining remission with baseline 1‐ and 3‐year DFS estimates. In addition to the overall CDFS, to assess the impact of EOC women's characteristics, 1‐ and 3‐year CDFS estimates were also calculated within the strata defined by age at diagnosis, marriage status, parity, stage, and histology.

All the statistical analyses were performed using the R software (version: 3.6.2) and GraphPad Prism software (version: 6.07). A *p*‐value of ≤.05 was considered statistically significant.

## 3 RESULTS

After applying the exclusion criteria to a total of 600 EOC patients who were evaluated, 335 patients were considered for the analysis. The mean ± SD age at diagnosis of the women was 48.2 ± 13.0 years (range: 18‐80 years) and 295 patients (88.1%) were married. The majority of the patients had a parity of 2‐5 (41.8%), followed by >5 parity (28.6%), nulliparous (20.9%), and 1 parity (8.7%).

**FIGURE 1:**
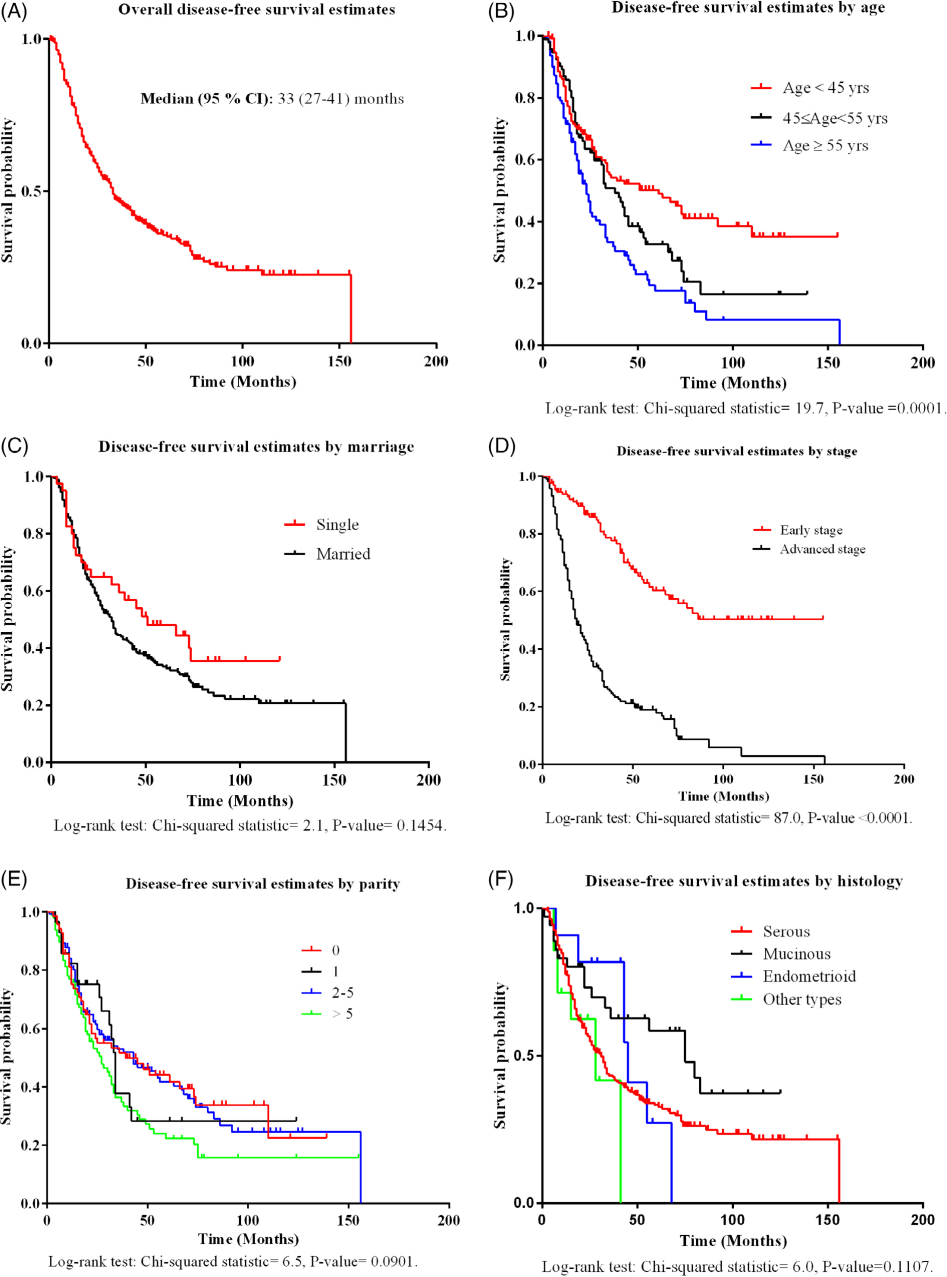
The nonparametric Kaplan–Meier survival curves of overall disease‐free survival (A) according to the categories of age (B), marriage status (C), stage (D), parity (E), and histology (F). The log‐rank test statistic and the associated *p*‐value are also shown for the comparison of various survival curves

The median (95% CI) OS was 3.58 (3.00‐4.17) years (ranging from 0.25 to 13.33 years). About 61.2% of the women were diagnosed with recurrent EOC, and 38.8% of them were still alive at the termination of the study. The median (95% CI) DFS for the women was 2.75 (2.25‐3.42) years (range: 0.08‐13.00 years). One‐, 2‐, 3‐, 4‐, 5‐, 7‐, and 10‐year traditional DFS rates (95% CI) for the EOC patients were calculated as 81.1% (76.4‐84.9%), 59.6% (53.9‐64.8%), 47.0% (41.2‐52.7%), 40.2% (34.4‐46.0%), 35.5% (29.7‐41.3%), 25.6% (19.8‐31.8%), and 21.8% (15.6‐28.7%), respectively (Figure 1A). The descriptive analyses demonstrated that 32.5%, 5.7%, 47.2%, and 14.6% of the study population had been diagnosed with stages I, II, III, and IV EOC, respectively. 53.0%, 6.7%, 32.9%, and 7.4% of the women who survived 5 years without recurrence had stages I, II, III, and IV EOC, respectively. Moreover, 81.8%, 10.7%, 3.3%, and 4.2% of the patients were diagnosed with serous epithelial, mucinous, and endometrioid carcinomas as well as other types of tumor (i.e., Brenner, undifferentiated carcinoma, and clear cell carcinoma) at baseline, respectively, while the EOC women who survived 5 years without recurrence were 76.5%, 15.4%, 3.4%, and 4.7% in the above‐mentioned histological subgroups, respectively.

Using the nonparametric log‐rank test analysis, the age of >55 years at diagnosis led to lower TDFS estimates (*p* = .0001) and women with ESEOC had significantly higher TDFS estimates than those with ASEOC (*p* < .0001). Moreover, the TDFS curves were not statistically significant in terms of marriage status (*p* = .1454), different histological subtypes (*p* = .1107), and child‐bearing or parity (0.0901) (Figure 1).

Landmark analyses were applied based on the multiple covariate Cox‐adjusted PH regression (Table 1) to establish the impact of various factors on the DFS at baseline and five subsequent years of remission. At baseline, the data analysis of all the 335 eligible patients demonstrated that a higher stage tumor (II: HR [95% CI]: 2.91 [1.40‐6.04], *p* = .004; III: HR [95% CI]: 5.20 [3.39‐7.99], *p* < .001; IV: HR [95% CI]: 6.46 [3.96‐10.56], *p* < .001) and endometrioid histology (HR [95% CI]: 2.59 [1.14‐5.89], *p* = .023) were associated with a higher risk of recurrence when compared to stage I and the other histological subtypes, respectively. For the 1‐ to 5‐year landmark time points, the DFS was evaluated from the specified landmark time point, and only EOC women who were still disease‐free at that landmark time point were considered in the analyses. The two previous factors (a higher stage tumor and endometrioid carcinoma) remained significant predictive factors of the DFS during five subsequent years of remission. Moreover, aging was also associated with a higher risk of recurrence at 1‐ and 3‐year landmark time points. This study showed that the hazard of recurrence for older women with the age at diagnosis of ≥55 years was approximately twice and three times more than that of young women aged <45 years at 1‐ and 3‐year landmark time points, respectively (1‐year: HR [95% CI]: 1.70 [1.01‐2.87], *p* = .047; 3‐year: HR [95%CI]: 3.71 [1.35‐10.20], *p* = .011).

**TABLE 1:** The hazard ratios (95% CIs) for recurrence using landmark analysis based on the multiple covariate Cox‐adjusted regression at baseline and the 1st, 2nd, 3rd, 4th, and 5th years of remission

		Baseline (*N* = 335)				1st year of remission (*N* = 264)			
Prognostic factor		N	E	HR (95% CI)	*p*	N	E	HR (95% CI)	*p*
**Age at diagnosis (year)**	<45	133	68	Reference	‐	106	41	Reference	‐
	45‐55	97	59	1.06 (0.73‐1.54)	.759	84	46	1.43 (0.91‐2.25)	.121
	>55	105	78	1.45 (0.97‐2.18)	.072	74	47	1.70 (1.01–2.87)	.047*
**Marriage status**	Single	40	23	Reference	‐	30	13	Reference	‐
	Married	295	182	1.20 (0.63‐2.29)	.575	234	121	1.52 (0.66‐3.50)	.324
**Parity**	0	70	41	Reference	‐	53	24	Reference	‐
	1	29	17	0.96 (0.49‐1.88)	.897	24	12	1.06 (0.46‐2.44)	.900
	2–5	140	80	0.73 (0.43‐1.22)	.231	114	54	0.68 (0.35‐1.33)	.262
	>5	96	67	0.82 (0.47‐1.42)	.469	73	44	0.80 (0.39‐1.62)	.534
**Stage**	I	109	36	Reference	‐	104	31	Reference	‐
	II	19	10	2.91 (1.40–6.04)	.004*	16	7	2.66 (1.12‐6.31)	.026*
	III	158	115	5.20 (3.39–7.99)	<.001*	112	69	4.54 (2.77‐7.44)	<.001*
	IV	49	44	6.46 (3.96–10.56)	<.001*	32	27	5.67 (3.18‐10.13)	<.001*
**Histology**	Serous	274	174	Reference	‐	214	114	Reference	‐
	Mucinous	36	17	1.00 (0.60‐1.68)	.993	30	11	0.83 (0.44‐1.59)	.581
	Endometrioid	11	7	2.59 (1.14–5.89)	.023*	10	6	3.04 (1.21‐7.62)	.018*
	Other types^a^	14	7	2.06 (0.95‐4.45)	.066	10	3	2.05 (0.63‐6.64)	.232

		2nd year of remission (*N* = 206)				3rd year of remission (*N* = 174)			
Prognostic factor		N	E	HR (95% CI)	*p*	N	E	HR (95% CI)	*p*
**Age at diagnosis (year)**	<45	91	26	Reference	‐	78	13	Reference	‐
	45–55	64	26	1.62 (0.89‐2.95)	.115	54	16	2.48 (1.07‐5.73)	.033*
	>55	51	24	1.98 (0.95‐4.15)	.068	42	15	3.71 (1.35–10.20)	.011*
**Marriage status**	Single	26	9	Reference	‐	24	7	Reference	‐
	Married	180	67	0.92 (0.24‐3.51)	.903	150	37	0.66 (0.13‐3.36)	.618
**Parity**	0	41	12	Reference	‐	38	9	Reference	‐
	1	22	10	3.18 (0.84‐12.06)	.088	14	2	1.09 (0.10‐12.35)	.946
	2–5	90	30	0.97 (0.28‐3.29)	.959	81	21	0.94 (0.21‐4.24)	.933
	>5	53	24	1.28 (0.35‐4.72)	.711	41	12	0.97 (0.18‐5.08)	.971
**Stage**	I	98	25	Reference	‐	92	19	Reference	‐
	II	14	5	3.84 (1.31‐11.24)	.014*	12	3	4.00 (0.93‐17.26)	.062
	III	73	30	3.70 (1.96‐7.01)	<.001*	56	13	2.46 (1.01‐6.00)	.047*
	IV	21	16	6.40 (3.07‐13.33)	<.001*	14	9	5.95 (2.34‐15.13)	<.001*
**Histology**	Serous	161	61	Reference	‐	133	33	Reference	‐
	Mucinous	27	8	0.86 (0.39‐1.91)	.712	24	5	0.64 (0.22‐1.84)	.403
	Endometrioid	9	5	5.00 (1.69‐14.78)	.004*	9	5	7.65 (2.31‐25.32)	.001*
	Other types^a^	9	2	4.20 (0.92‐19.16)	.064	8	1	38.68 (1.92‐777.6)	.017*

		4th year of remission (*N* = 158)				5th year of remission (*N* = 149)			
Prognostic factor		N	E	HR (95% CI)	*p*	N	E	HR (95% CI)	*p*
**Age at diagnosis (year)**	< 45	76	11	Reference	‐	74	9	Reference	‐
	45–55	46	8	1.58 (0.56‐4.41)	.386	43	5	1.06 (0.25‐4.51)	.933
	>55	36	9	2.18 (0.63‐7.56)	.220	32	5	1.27 (0.23‐7.10)	.785
**Marriage status**	Single	21	4	Reference	‐	20	3	Reference	‐
	Married	137	24	1.00 (0.17‐5.96)	.996	129	16	1.12 (0.14‐8.86)	.917
**Parity**	0	35	6	Reference	‐	34	5	Reference	‐
	1	12	0	NA	NA	12	0	NA	NA
	2–5	75	15	0.81 (0.17‐3.82)	.788	71	11	0.67 (0.12‐3.74)	.648
	>5	36	7	0.67 (0.10‐4.37)	.677	32	3	0.20 (0.01‐3.21)	.257
**Stage**	I	84	11	Reference	‐	79	6	Reference	‐
	II	11	2	7.41 (1.14‐48.36)	.036*	10	1	33.20 (2.14‐515.95)	.012*
	III	51	8	2.93 (0.92‐9.39)	.070	49	6	6.59 (1.33‐32.74)	.021*
	IV	12	7	9.58 (3.11‐29.54)	<.001*	11	6	42.61 (8.59‐211.38)	<.001*
**Histology**	Serous	121	21	Reference	‐	114	14	Reference	‐
	Mucinous	24	5	1.05 (0.32‐3.43)	.938	23	4	2.25 (0.49‐10.25)	.295
	Endometrioid	6	2	12.07 (2.06‐70.68)	.006*	5	1	96.39 (6.54‐1421.36)	.001*
	Other types^a^	7	0	NA	NA	7	0	NA	NA

Abbreviations: CI, confidence interval; E, number of desired events in each category; HR, hazard ratio; *N*, number of observations in each category; NA, not able to compute because no event was observed or the sample size was small in the desired year after remission; *p*, *p*‐value.

^a^ Other types include clear cell carcinoma, Brenner tumor, undifferentiated carcinoma.

* *p*‐value of ≤.05 was considered significant.

**FIGURE 2:**
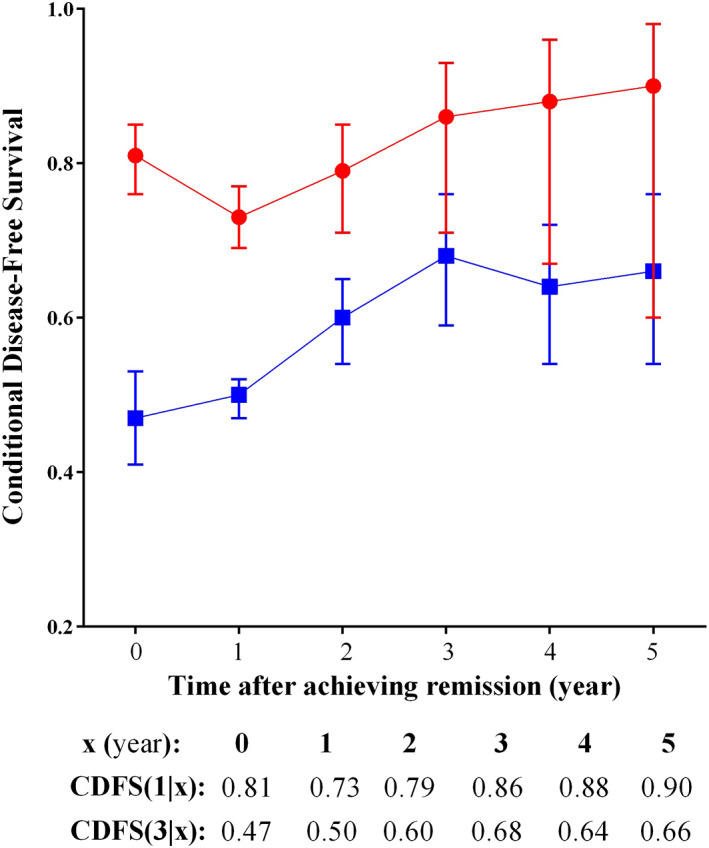
One‐ and 3‐year conditional disease‐free survival (CDFS) estimates. The vertical bars indicate the 95% confidence intervals (CIs) of the corresponding point estimates (1‐year CDFS (red) is represented by CDFS (1|x) and 3‐year CDFS (blue) is shown by CDFS (3|x))

At baseline, the 1‐ and 3‐year DFSs were 81.1% and 47.0%, respectively, and decreased over time. The 1‐ and 3‐year CDFS estimates for EOC women are depicted in Figure 2. Based on the concept of CDFS, the probabilities of staying disease‐free for an additional 1 year were 73%, 79%, 86%, 88%, and 90%, respectively, given that the patient has already been in remission for x years (i.e., CDFS (1|x)) and x = 1, 2, 3, 4, and 5. Moreover, the probability of surviving an additional 3 years without recurrence, conditioned on having already survived x years after remission (i.e., CDFS (3|x)) when x = 1, 2, 3, 4, and 5 improved to 50%, 60%, 68%, 64%, and 66%, respectively (Figure 2).

**FIGURE 3:**
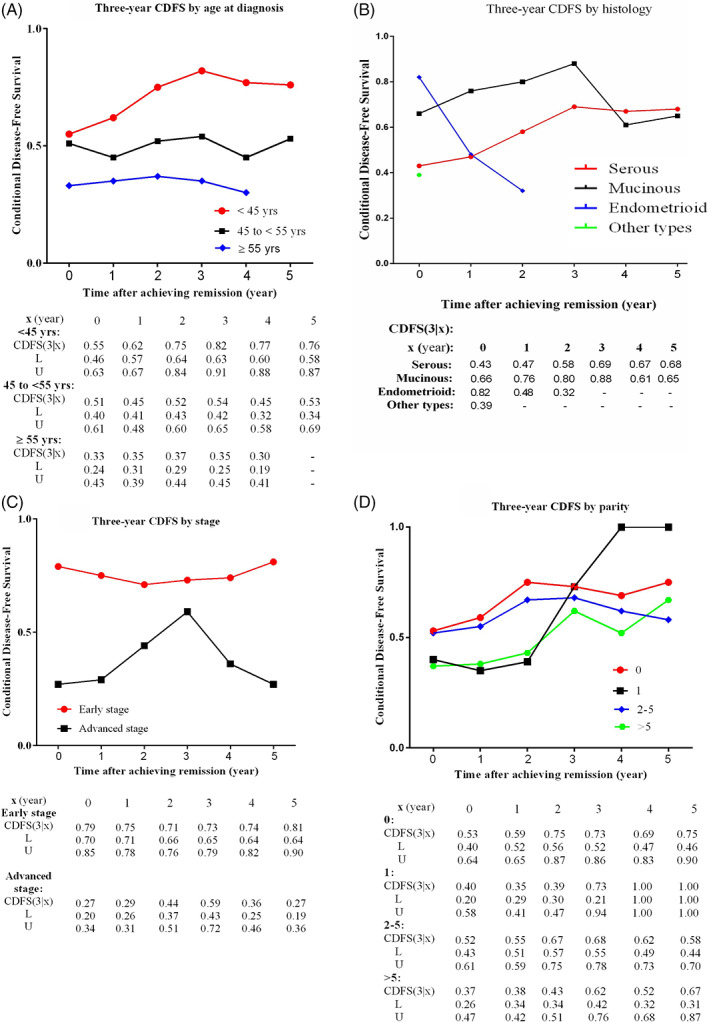
The conditional probabilities of surviving an additional 3‐year disease‐free period (CDFS (3|x)) and the corresponding 95% confidence intervals (L = lower bound; U = upper bound) at a particular prediction time by age (A), histology (B), stage (C), and parity (D)

Figure 3 depicts 3‐year CDFS rates stratified by age at diagnosis, histology, stage, and child‐bearing (parity). The missing values for some CDFSs (3|x) are due to the limitations of subgroup size. For instance, in Figure 3, the probability of staying disease‐free for an additional 3 years given that an EOC woman with mucinous carcinoma has already been in remission for 4 years is equal to 0.61, that is, CDFS (3|4) = 0.61. In contrast to serous and mucinous histology subtypes, the 3‐year CDFS of the patients with endometrioid carcinoma gradually decreased over time (see Figure 3B).

**FIGURE 4:**
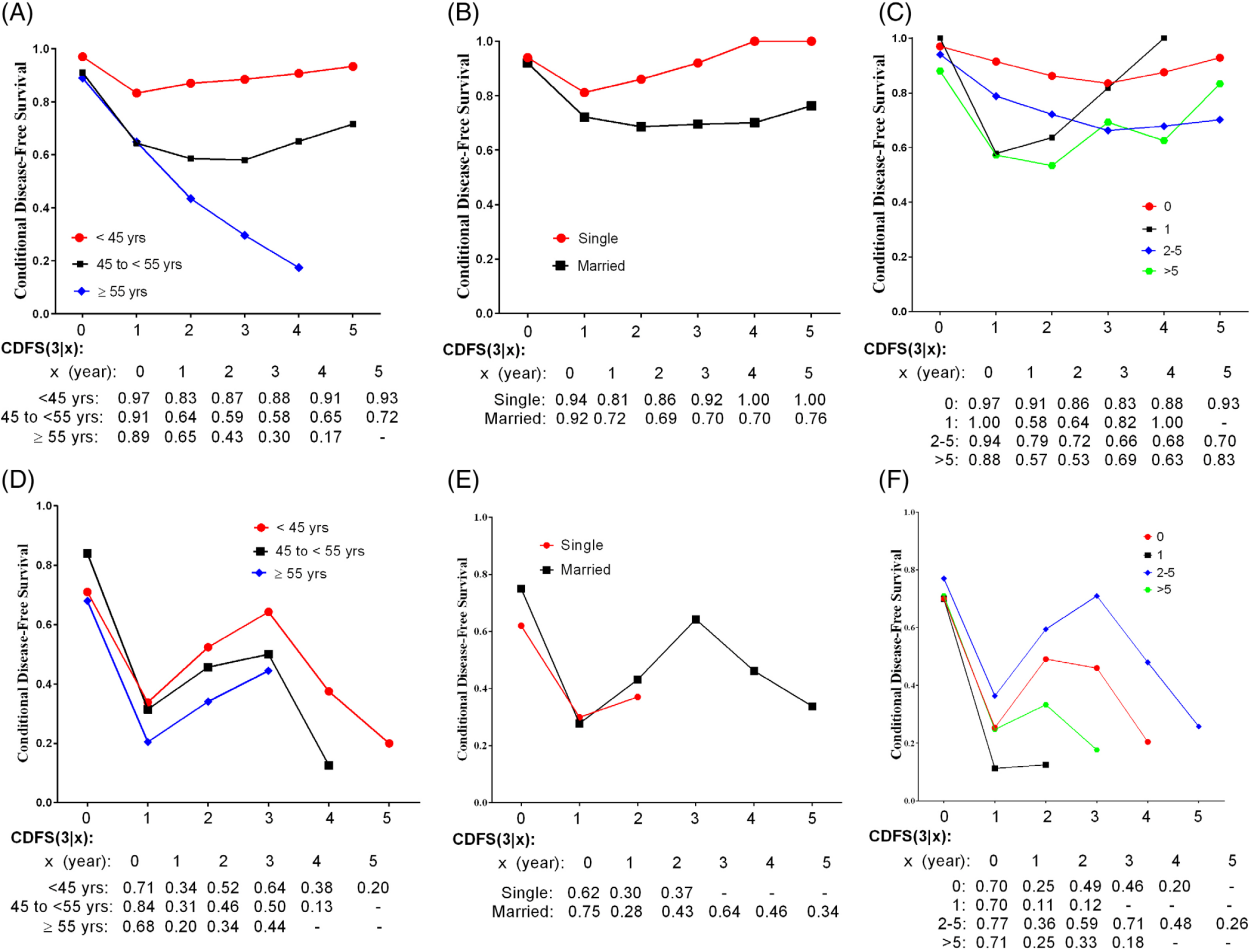
Three‐year conditional disease‐free survival (CDFS) estimates by stage subgroup analyses: early‐stage (A: age, B: marriage status, and C: parity) and advanced stage (D: age, E: marriage status, and F: parity)

However, the largest improvements in 3‐year CDFS estimates were seen for young women with one child and those diagnosed with advanced‐stage disease (Figure 3). The subgroup analyses based on disease stage (early/advanced) indicated that the 3‐year CDFS of older women in the early stage (Figure 4A) gradually decreased during the 3 years of follow‐up.

## 4 DISCUSSION

This study was the first study which assessed CDFS among Iranian women with EOC. It mainly highlighted the prognostic determinants having a great impact on DFS. Furthermore, the pivotal factors that could change the risk of recurrence were determined. It was found that CDFS estimates differed with the histology of disease and age at diagnosis. Although the patients with ESEOC had higher CDFS estimates compared to those with ASEOC, there was a gradual decrease in the 3‐year CDFS of those with ESEOC. However, the 3‐year CDFS showed a trend for an increase in the ASEOC group. The 3‐year CDFS of the patients with the age of ≥55 years was significantly lower than that of younger patients. Subgroup analysis based on the stage of disease (Figure 4) demonstrated that age at diagnosis was an important prognostic factor for ESEOC and significantly influenced the 3‐year CDFS estimates.

Significant relationships between the risk of recurrence and the assessed patient characteristics including age at diagnosis, stage of disease, and histology were observed at baseline. These findings are in line with some previous studies which indicated the role of these factors as predictors of overall or DFS estimates.^12^ Some predictors of survival proposed in past surveys are the stage of disease,^12^, ^17^ parity and histology,^3^ family history,^18^ the total number of received chemotherapy cycles,^19^ pretreatment CA‐125,^20^ and the number of chemotherapy cycles before normalization of CA‐125.^21^ In this study, the effects of the characteristics of the patients who had already been in remission for 1 to 5 years on their subsequent TDFS and CDFS were assessed as well. The results demonstrated that the stage of disease and histology remained as predictive factors of 5‐year DFS.

A previous study found initial differences in the DFS of OC patients at the time of remission among stage, age at diagnosis, grade, and histology groups which diminished over time.^12^ However, the differences in 3‐year CDFS estimates based on age at diagnosis, histology, and stage remained high and did not diminish over time in the present study. Therefore, it can be inferred that each of these characteristics should be considered separately for each patient. Regarding age at diagnosis, 3‐year CDFS was at the lowest level in patients over 55 years of age compared to the other two age groups and remained approximately flat over time. Besides, 3‐year CDFS increased in women with the age of fewer than 45 years for 3 years after initial treatment, and then it slightly decreased. This finding demonstrated that age at diagnosis can be used as a prognostic factor for the prediction of the 3‐year recurrence. It was also found that the probability of recurrence did not decrease over time in patients over 55 years of age.

According to the results of this study, the 3‐year CDFS of ESEOC patients was different from that of the ASEOC patients. The CDFS of the ESEOC patients diminished in the first 2 years after achieving remission and then gradually increased. These findings indicate that ESEOC patients need accurate surveillance during the first 2 years after achieving remission. Although the baseline value of the 3‐year DFS of the ASEOC patients (27%) was markedly lower than that of the ESEOC patients (79%), the 3‐year CDFS of the ASEOC patients increased in the first 3 years after remission achievement and then suddenly decreased with time. It can be concluded that ASEOC women need more accurate surveillance after the first 3 years of follow‐up. Considering the observed differences in the CDFS of early and advanced stage EOC in this study, subgroup analyses were done based on the stage of disease, and the effects of various factors were also assessed.

The 3‐year CDFS of the ESEOC patients analyzed based on different age groups demonstrated a gradual increase in 1 year after remission in patients with the age at diagnosis of <45 years. This indicates that the probability of tumor recurrence in this age group decreased for 5 years after achieving remission. However, in the ESEOC patients with the age at diagnosis of >55 years, CDFS gradually decreased during the 4 years following remission which demonstrated that the probability of tumor recurrence in this age group remained high even after 4 years. Hence, regular follow‐up visits are required for this age group. The 3‐year CDFS of the ASEOC patients followed an almost similar trend in all the three age groups and showed an increase from 1 to 3 years after the achievement of remission and a decrease after 3 years. It can be inferred that the stage of the disease is a more important prognostic determinant in ASEOC patients than the age at diagnosis.

Different prognostic factors have been mentioned in younger and elderly patients in a previous study. The FIGO stage and standard primary treatment were independent determinants in younger women, whereas performance status was identified as the independent determinant in elderly women.^22^ On the other hand, another study showed higher survival rates for very young EOC patients compared to young or older groups.^23^ This result might be related to the medical and physiological conditions which might be present in older EOC patients and require special attention in planning treatment. Therefore, polypharmacy therapy, disability, and multimorbidity in older EOC women could lead to poor prognosis, mortality, and surgical complications.^24^, ^25^ A progressive decline of organ functions and the increased prevalence of chronic diseases that can cause pharmacodynamic and pharmacokinetic changes of drugs are more common in older patients than in younger ones. Therefore, the difficult adaptation of old patients to standard treatments can justify the worse prognosis for patients with the age of >55 years in the present study compared to the other age group patients.^26^ However, a previous study demonstrated that the mortality and morbidity percentages were the same across younger and older patients who were equally debulked.^27^ Another study also found that age was not an independent prognostic factor for either OS or DFS among EOC women and performance status affected the treatment outcome for elderly patients.^22^ In general, there is still controversy about the prognostic effect of age in EOC women.

The subgroup analysis based on histology indicated that the 3‐year CDFS of the EOC women with endometrioid carcinoma gradually decreased during the 3 years after remission. This result was in contrast to the 3‐year CDFS of patients with serous carcinoma and mucinous carcinoma (Figure 4B). Based on the results of the present study, endometrioid carcinoma has the worst prognosis among the histological types of EOC. Therefore, patients with endometrioid carcinoma need regular and more intense follow‐up examinations. Previous research also found an improvement in the OS rate for EOC women with clear cell and endometrioid carcinomas and mucinous cystadenocarcinoma.^18^ The OS of stage IV EOC women with mucinous and clear cell subtypes was significantly lower than that of women with other histological subtypes in another previous work as well.^28^ However, due to the few numbers of endometrioid subtypes in the present study (11 cases out of 335), it was not possible to compare the results of this study with those of previous studies directly.

A study conducted on EOC patients indicated that more than one parity improved OS in ASEOC patients.^3^ However, the results of the current study did not show any relationship between the CDFS of EOC women and their parity.

One of the goals of follow‐up is the early detection of disease recurrence. However, there is no consensus on whether increased surveillance for recurrent disease can necessarily improve OS.^18^, ^29^ Besides, no clear guidelines are available on the type and frequency of follow‐up care. One of the earliest indicators of disease recurrence in EOC patients is a more than twice rise of the upper limit of normal CA‐125. However, the early treatment of tumor recurrence based on the increased level of CA‐125 did not demonstrate a significant survival benefit.^10^

The results of landmark analyses showed that age at diagnosis and histological type were predictive of subsequent DFS after 1 to 5 years of remission. These were in line with the results of a previous study which found that the main prognostic factor for the DFS of the EOC patients was tumor histology [28]. Moreover, it has been reported that age at diagnosis, the results of primary surgery and second‐look surgery, stage of disease, optimal debulking surgery, and type of chemotherapy influenced the 5‐year progression‐free survival of the EOC patients.^30^

This study had some limitations. The results of the present study should be confirmed in future studies with larger sample sizes. Moreover, in future studies, the amount of residual tumor after cytoreductive surgery should be considered due to its possible role in CDFS. Since the patient's response to different chemotherapy regimens depends on the type of tumor, future studies should also be conducted to assess the outcome of the disease course as a function of tumor histology.

## 5 CONCLUSIONS

It was concluded that CDFS for EOC patients was different based on age at diagnosis, stage of the tumor, and histology. Age at diagnosis, defined by a cut‐off of 55 years, was a prognostic factor for the CDFS of EOC women. Moreover, patients with ASEOC (stages III and IV) and endometrioid histology had poorer CDFSs compared to those with ESEOC (stages I and II) and other histological types of tumors. In the ESEOC patients with the age at diagnosis of >55 years, CDFS gradually decreased during the 3 years after remission. These highlight the need for more tailored management and continuous care during the surveillance of ESEOC patients with the age at diagnosis of >55 years and endometrioid histology.

## CONFLICT OF INTEREST

The authors have stated explicitly that there are no conflicts of interest in connection with this article.

## AUTHOR CONTRIBUTIONS

*Data curation; formal analysis; investigation; methodology; software; supervision; visualization; writing‐original draft; writing‐review & editing*: Vahid Ebrahimi. *Conceptualization; data curation; formal analysis; investigation; methodology; software; visualization; writing‐original draft; writing‐review and editing*: Abolfazl Khalafi‐Nezhad*. Conceptualization; data curation; investigation; methodology; software; supervision; visualization; writing‐original draft; writing‐review & editing*: Fatemeh Ahmadpour. *Conceptualization; data curation; formal analysis; funding acquisition; investigation; methodology; resources; software; writing‐original draft; writing‐review & editing*: Zahra Jowkar.

## ETHICS STATEMENT

The study protocol was approved by the Ethics Committee for Research of Shiraz University of Medical Sciences (Protocol# IR.SUMS.REC.1393.8910) and was conducted in compliance with the Declaration of Helsinki. All patients had given written informed consent to allow the use of their personal and clinical data retrospectively.

## PATIENT CONSENT

All study participants, or their legal guardian, provided written informed consent prior to study enrollment. Besides, the patients who did not provide signed informed consent for the analysis of their medical records were excluded.

## Data Availability

The data used to support the findings of the present study are available from the corresponding author upon request.

## APPENDIX A.

### Definition of conditional disease‐free survival

Conditional disease‐free survival (CDFS) estimates are directly computed from nonparametric Kaplan–Meier (NPKM) disease‐free survival estimates. Let $\hat{S}\left(t\right)$ demonstrate the NPKM disease‐free survival estimate at time t. CDFS is defined as the probability of staying disease‐free for an additional number of years (t_2_) given that a patient has already been in remission for t_1_ number of years. It can be calculated using the following equation^11^, ^31^, ^32^:

$$C\hat{\mathrm{DF}}S\left(t_{2}|t_{1}\right)=\frac{\hat{S}\left(t_{1}+t_{2}\right)}{\hat{S}\left(t_{1}\right)}$$

For instance, to calculate the 2‐year CDFS estimate for patients who had already been in remission for 2 years (i.e., $C\hat{\mathrm{DF}}S\left(22\right)$), the 4‐year NPKM disease‐free survival estimate ($\hat{S}\left(4\right)$) was divided by the 2‐year NPKM disease‐free survival estimate ($\hat{S}\left(2\right)$):

$$C\hat{\mathrm{DF}}S\left(22\right)=\frac{\hat{S}\left(2+2\right)}{\hat{S}\left(2\right)}=\frac{\hat{S}\left(4\right)}{\hat{S}\left(2\right)}$$

Confidence intervals (CIs) can be computed around CDFS estimates using the Greenwood formula for the estimation of CIs in unconditional survival.^12^ The CIs are based on a variation of the log–log transformation proposed by Prentice and Kalbfleisch and known as the exponential Greenwood formula.^13^ Using the exponential Greenwood formula, the traditional DFS estimate is substituted by the CDFS estimate in constructing the CI. The 95% CI for CDFS can be defined as:

$${\left[C\hat{\mathrm{DF}}S\left(t_{2}|t_{1}\right)\right]}^{\exp\left\{\pm 1.96\times \sqrt{\hat{M}\left(t_{2}|t_{1}\right)}\right\}}$$

where

$$\hat{M}\left(t_{2}|t_{1}\right)=\frac{1}{{\left[\log\left(C\hat{\mathrm{DF}}S\left(t_{2}|t_{1}\right)\right)\right]}^{2}}\sum_{j:t_{1}\leq \tau_{j}\leq t_{2}}\frac{d_{j}}{\left(r_{j}-d_{j}\right)r_{j}}$$

and

τ_j_ = distinct event time j

r_j_ = number at risk at event time j

d_j_ = number of failures at event time j.

### Conditional disease‐free survival at baseline

The estimation of the CDFS at baseline can be calculated as:

$$C\hat{\mathrm{DF}}S\left(t_{2}|0\right)=\frac{\hat{S}\left(0+t_{2}\right)}{\hat{S}\left(0\right)}=\frac{\hat{S}\left(t_{2}\right)}{1}=\hat{S}\left(t_{2}\right)$$

In fact, $\;C\hat{\mathrm{DF}}S\left(t_{2}|0\right)$ is equal to the estimation of the survival function at survival time t_2_, that is, $\hat{S}\left(t_{2}\right)$. Therefore, CIs around CDFS (t_2_| 0) = S(t_2_) can be calculated using the Greenwood formula in the unconditional survival analysis.^12^ The 95% CI formula for CDFS (t_2_| 0) = S(t_2_) has a general form as:

$$\hat{S}\left(t_{2}\right)\pm 1.96\sqrt{\hat{\mathrm{var}}\left(\hat{S}\left(t_{2}\right)\right)}$$

Greenwood's formula for $\hat{\mathrm{var}}\left(\hat{S}\left(t_{2}\right)\right)$ is given by:

$$\hat{\mathrm{var}}\left(\hat{S}\left(t_{2}\right)\right)={\left[\hat{S}\left(t_{2}\right)\right]}^{2}\sum_{j:0\leq \tau_{j}\leq t_{2}}\frac{d_{j}}{\left(r_{j}-d_{j}\right)r_{j}}$$

where.

τ_j_ = distinct event time j

r_j_ = number at risk at event time j

d_j_ = number of failures at event time j.
